# Relationships between Brain Structure and Metabolic Changes in Schizophrenia Patients Treated with Olanzapine: A Voxel-Based Morphometric Study

**DOI:** 10.1155/2011/862350

**Published:** 2011-05-25

**Authors:** Genevieve Letourneau, Lahcen Ait Bentaleb, Benjamin Stip, David Luck, Emmanuel Stip

**Affiliations:** ^1^Department of Psychiatry, University of Montreal, Montreal, QC, Canada H3C 3J7; ^2^Fernand-Seguin Research Center, Montreal, QC, Canada H1N 3V2; ^3^Department of Psychiatry, Louis H. Lafontaine Hospital, Montreal, QC, Canada H1N 3M5; ^4^Department of Psychiatry, Pavillon Albert-Prevost, Montreal Sacre-Coeur Hospital, Montreal, QC, Canada H4J 1C5

## Abstract

*Introduction*. Second-generation antipsychotics treatment is associated with weight gain and metabolic disturbances. Although much research has been done on the topic, the precise mechanisms underlying such side effects are still not well understood. *Method*. We followed over 16 weeks a group of 17 schizophrenia patients who were treated with olanzapine and monitored biometric, clinical, and metabolic data, including ghrelin and leptin levels. All patients had a structural cerebral magnetic resonance imaging examination during the first week of their followup and at the end of the study. *Results*. We found positive and negative significant correlations between grey matter volumes of several brain regions and variations of body weight as well as of ghrelin and leptin levels. The right frontal operculum, bilateral precuneus, and bilateral hippocampal regions were found to be significantly associated with those changes. *Conclusion*. Our results suggest associations between brain structure and metabolic variations in schizophrenia patients taking olanzapine.

## 1. Introduction

Second-generation antipsychotics, such as olanzapine and clozapine, are associated with weight gain [1] and various metabolic disturbances [2]. These adverse effects are associated with a significant increase of morbidity among the affected patients [3]. Metabolic changes have been associated with an important increase of appetite [4] but might as well be secondary to other mechanisms, all of which are still poorly understood despite the important amount of research that has already been produced on the topic. It has also been described that weight gain secondary to antipsychotic therapy was also associated with better clinical response [5], which can raise new questions and new hypothesis as for the mechanisms of weight gain.

Over the last years, the involvement of neuropeptides and hormones, such as ghrelin and leptin, in the appetite and eating behavior mechanisms has been studied. Ghrelin is an orexigenic signalling hormone, especially produced in the stomach and released in the blood stream, circulating in the whole body [6]. Ghrelin secretion is regulated by food intake, and its concentration rises during fasting and lowers after meals [7]. The rise of ghrelin levels could play a role in the meal initiation [8]. Levels of plasma ghrelin show a negative correlation with BMI and are high in thin or anorexic people and low in obese people [9]. Ghrelin is the endogenous ligand for a Growth Hormone secretagogue receptor (GHS-R), which means that it stimulates Growth Hormone (GH) release. Ghrelin and GHS-R mRNAs have been found in the heart and the pancreas, suggesting their role in cardiovascular and metabolic processes. Ghrelin is also found in the central nervous system. In fact, ghrelin-receptive neuronal cells were discovered in the hypothalamic arcuate nucleus, the hippocampus, and the ventrotegmental area (VTA). It is thought that ghrelin stimulates GH release through direct stimulation on the pituitary gland, in a dose-dependent way. Ghrelin stimulates the expression of the NPY neuropeptide and agouti-related protein in the arcuate nucleus of the hypothalamus, which are appetite-stimulating molecules [10]. Ghrelin-containing neurons could also modulate the activity of proopiomelanocortin (POMC) in the arcuate nucleus, leading to a decrease of melanocortin peptides and an increase of food intakes. Moreover, ghrelin, when administered in the VTA, increases locomotor activity and dopamine levels in the nucleus accumbens. Low levels of ghrelin have been associated with insulin resistance, hypertension, and higher prevalence of type 2 diabetes in a general, nonpsychiatric, population [11].

Leptin is an anorexigenic hormone produced by the adipocytes, whose levels reflect the amount of fat stored as well as the energy imbalance [12]. Levels of leptin get higher when people overeat and lower when people fast. Leptin activates receptors that are found in many areas of the brain, specially the arcuate nucleus of the hypothalamus, where it reduces the expression of hypothalamic neuropeptides, such as the neuropeptide Y and agouti-related peptide, resulting in a decrease of appetite. Leptin also activates proopiomelanocortin (POMC) cells in the hypothalamus [13], which increases the release of melanocortin peptides inhibiting food ingestion and regulating metabolism [14]. It has also been described that leptin is also active in the VTA [15], decreasing food intake and locomotor activity [16]. 

Many articles have described the variations of fasting ghrelin and leptin levels in schizophrenic patients on atypical antipsychotic treatment. A review article [17] reported that leptin levels were rising early during atypical antipsychotic treatment (mostly olanzapine and clozapine) but no alteration of leptin levels was associated with conventional antipsychotics. Ghrelin levels showed a biphasic evolution, decreasing in the first few weeks of treatment then rising when treatment was taken over a longer period. The authors of the review pointed out that no correlation had been done between the weight gain and the variations of the neuropeptides levels. In fact, one recent study had suggested that leptin changes, when the weight gain confusion factor had been removed, were not significant [18]. 

Associations between leptin levels and cerebral structures in a nonpsychiatric population have also been described, and positive correlations were found between leptin levels and cerebellum and inferior temporal gyrus, while negative correlations were found between leptin levels and the inferior frontal operculum, the postcentral gyrus, and the putamen [19]. These findings were similar to those found in cerebral structural studies on obesity [20]. 

Recent research has shown that antipsychotics could induce cerebral structural modifications [21–23] in schizophrenia patients and reports emerged describing different modifications whether patients were on typical versus atypical antipsychotic treatment, and correlations have been established between clinical improvements and cerebral changes while patients were taking antipsychotics [24].

No study has yet been done on associations between ghrelin levels and brain structures. No study either has yet examined the relationships between weight gain, ghrelin/leptin changes, and cerebral structural changes for patients taking atypical antipsychotics. We were interested in investigating whether cerebral structural modifications could play a role in the appetite mechanisms and the subsequent weight gain that has been described in schizophrenic patients treated with olanzapine.

## 2. Method

The present study investigates the relationships between cerebral volumes and metabolic changes, more specifically body weight modifications and variations of fasting ghrelin and leptin levels. We recruited a cohort of patients suffering from schizophrenia according to DSM-IV criteria. Patients were started on olanzapine when entering the study. They should not have taken olanzapine for at least six months before entering the study, but other antipsychotics were allowed during that time period. That being said, olanzapine was the only allowed antipsychotic medication during the study. Other types of psychiatric medications were allowed, such as antidepressants and benzodiazepines.

Written informed consent was obtained from all subjects after complete explanations of the study were provided. The study was approved by the Ethics Committee of Fernand Seguin Research Center, in Montreal, Canada.

Patients were followed for 16 weeks. A first magnetic resonance examination was scheduled during the first week of the study and repeated at the end of the followup, at week 16 or earlier. Biometrical data, such as body weight, body mass index (BMI), abdominal circumference, and blood pressure, were controlled at weeks 1, 2, 8, 12, and 16. At the same time, blood samples were taken and analysed for fasting glucose, cholesterol, triglycerides, leptin, and ghrelin levels. All blood samples were taken in the morning, after an overnight fast. Fasting leptin and ghrelin were analysed using radioimmunoassay techniques for human leptin (HL-81K) and total ghrelin (GHRT-89HK) kits developed by LINCO Research (Mo, USA).

Clinical followup also took place at weeks 1, 2, 8, 12, and 16. PANSS (Positive and Negative Symptoms Scale) [25] and CDSS (Calgary Depression Scale for Schizophrenia) [26] were then done with all patients by a single investigator. TFEQ (Three-Factor Eating Questionnaire) [27] was chosen to monitor appetite and eating behaviour changes in patients and was also to be performed during those follow-up meetings. 

## 3. Population

Twenty-four patients (nineteen men and five women), fulfilling the DSM-IV criteria for schizophrenia participated in the study. The mean (SD) age was 31, 38 (8.79) years (range: 21–51). The mean olanzapine dosage was 15, 42 (6.74) mg per day (range: 5–30). At the beginning of the study, all antipsychotic medications had to be discontinued while patients were started on olanzapine. Three patients were not taking any antipsychotics at the time of recruitment while 21 of them were taking one or more antipsychotic medications. Fifteen patients had been taking risperidone, 6 quetiapine, 1 clozaril, 2 haloperidol, and 1 perphenazine before entering the study. 

Twenty patients entered the study and passed a first pretreatment MRI but only seventeen of them passed a second posttreatment MRI. The reasons for dropping out were consent withdrawal (1) subject(s), incapacity to go through the examination (1) subject(s), and lack of observance to treatment (2) subject(s). One patient entered the study, could not complete the first MRI examinations because of discomfort during the session, but completed a second MRI. Two patients, who had been on olanzapine treatment for more than 16 weeks, were added to the cohort and they only passed a posttreatment MRI examination. 

While sixteen subjects took the treatment for 16 weeks before passing their second MRI, one subject had to discontinue treatment and passed an early posttreatment MRI (after six weeks), both because he presented excessive increase of appetite and weight gain. One patient had only one MRI at 11 weeks of treatment as he did not tolerate the first examination and had to discontinue treatment early as he also presented excessive appetite and weight gain. The results discussed in the present article concern those 17 patients. 

All subjects signed an informed detailed consent approved by the ethics committee before participating in the study.

### 3.1. MRI Acquisition

High-resolution T1-weighted 3D volume acquisition was acquired on a 3 Tesla System (Magetom Vision, Siemens Electric, Erlagen, Germany) by using a gradient echo pulse sequence (TE: 44 msecs, Flip: 120°, FOV: 250 mm, Matrix: 256 × 256, Voxel size: 2,0 × 2,0 × 2,0 mm^3^, Number of slices: 164 sagittal slices). 

### 3.2. VBM Procedure and Analyses

Voxel-Based Morphometry (VBM) was performed using Statistical Parametric Mapping 8 (SPM8) software package (http://www.fil.ion.ucl.ac.uk/spm/), running on Matlab 7.4 under Linux operating system. We used the Montreal Neurological Institute (MNI) template and a priori probability maps for gray matter, white matter, and cerebral spinal fluid were constructed. The T1 of each subject was spatially normalised to the MNI template to correct for differences in brain size and shape and facilitate intersubject averaging. We coregistered the subjects at time two on the subjects at time 1, to minimise the differences related to head position. Images from times 1 and 2 were then normalised again with the same normalisation matrix, modulated, and smoothed at 8 mm full-width at half-maximum isotropic Gaussian kernel before statistical analyses were performed. Paired *t*-tests were done to compare brains structure variations (between time 1 and 2) using the SPM-8 statistical tools. As well, 1 sample *t*-tests were done using SPM8 statistical tools to investigate the relationships between body weight, fasting leptin and ghrelin, and brain structures after a 16 weeks treatment with olanzapine.

## 4. Results

The group of seventeen patients (thirteen men, four women) who completed the study and passed two MRI examinations had a mean (SD) age was 32,82 (9,51) years. They had been on a mean daily olanzapine dosage of 16,18 (6,97) mg that they had taken for an average of 110,47 (18,56) days at the time they left the study.

A summary of the biometric, metabolic, and clinical variations of the 17 patients after the 16 weeks olanzapine treatment is shown in Table 1, including changes in body weight (kg), BMI (kg/m^2^), abdominal circumference (cm), fasting glucose (mmol/L), total cholesterol (mmol/L), triglycerides (mmol/L), ghrelin (pg/mL), leptin (ng/mL), and PANSS global scores.

Table 2 shows the correlations found between grey matter volumes after a 16 weeks olanzapine treatment and body weight changes observed in schizophrenia patients. In order to investigate how structural brain changes could associate with changes of fasting ghrelin and leptin, we did positive and negative correlations for fasting ghrelin and leptin level changes with the structural brain images after olanzapine treatment. Tables 3 and 4 summarise the correlations between grey matter volumes and fasting ghrelin/leptin levels changes observed in schizophrenia patients after 16 weeks of olanzapine treatment. 

## 5. Discussion

Significant positive correlations were found between body weight variations and the gray matter volume in the right superior parietal and right inferior frontal operculum regions. Superior parietal region is associated with visual processing and has been identified as particularly active among hungry individuals who were presented with pictures of food of high hedonic value. Interestingly, this region was also found less active among normal individuals after a period of overeating [28]. A functional study done in a nonpsychiatric population had demonstrated positive correlations between BMI and activations in different regions of the operculum in response to consummatory food reward [29]. The frontal operculum is part of the gustatory cortex and has been described as more active in obese people in response of anticipatory food intake [28]. Significant negative correlations between body weight variations and grey matter volumes were found in the left postcentral gyrus and left hippocampus, as well as bilateral precuneus and thalamus. Functional imaging studies have demonstrated increased neural activity in the postcentral gyrus of obese subjects [30, 31], as well as identified postcentral gyrus activations during food swallowing [32]. Moreover, the left postcentral gyrus has been described as significantly less dense in obese people, compared to lean subjects, suggesting its involvement in weight gain mechanisms. The hippocampus is part of the limbic region, which plays a role in the generation of affective responses to internal stimuli and receives projections from the orbitofrontal cortex, which integrates sensory and visceral afferents, motivating the individual to behave in a way that will alleviate hunger [33]. The hippocampus is a cerebral region where receptors for leptin, whose levels reflect the body fat storage, have been found [34] and where leptin-induced changes in dendritic morphology have been described [35]. A recent study had also demonstrated that leptin administration was associated with decreases of protein kinase b in hippocampal and cerebellar regions [36] and that high-fat diets were associated with higher hippocampal oxidative stress in animal models [37]. Precuneus region also plays a role in appetite control as lowering of activity in this region has been associated in functional studies with satiety. It has been also demonstrated that precuneus activity was inversely correlated with blood insulin variations in obese people only. Thalamus has also been demonstrated to play a role in hunger mechanism, as a relay point for perception and learning. 

Significant positive correlations were found between ghrelin variations and the fusiform, superior temporal, and frontal operculum regions. The frontal operculum region has already been described as part of the gustatory cortex and the fusiform cortex, in which activations have been found in appetite studies, was linked to the integration of visual appetising stimuli. Significant negative correlation was found in the caudate, which is interesting as well since functional studies have already associated weaker activation in the striatum with greater risk of overeating and weight gain [30] and have described initially decreasing levels of fasting ghrelin in patients treated with atypical antipsychotics. Recent research has suggested that ghrelin could promote and protect the dopaminergic nigrostriatal pathway [38] in which case the negative correlation finding would be surprising and rather reflect the global increase in the caudate related to the D2 antagonist effects on the nigrostriatal pathway while olanzapine-induced movement disorders are doserelated [39] and the average dosages were above 15 mg per day in our cohort.

 Significant positive correlations have been found between leptin changes and grey matter density in precentral regions, which have been associated with satiety in many functional studies. A negative significant correlation was found between leptin changes and grey matter in right middle frontal, an area which has been associated with behavioural aspects of appetite and in bilateral precuneus, an area where activations related to hunger have been described in functional neuroimaging studies [33]. Significant negative correlations have also been found in the hippocampus, a cerebral region with implications related to leptin levels that were discussed earlier. 

Some regions emerged as correlated with many metabolic parameters variations. The right frontal operculum grey matter volume was found positively correlated with weight change, and ghrelin level change, which makes sense since ghrelin is an orexigenic hormone, associated with meal initiation and feeding. It is interesting to relate this finding to other results from other structural studies in eating disorders that demonstrated significantly reduced volumes of the frontal operculum in anorexia nervosa patients compared to healthy controls [40]. The bilateral precuneus grey matter volume was found negatively correlated with weight change and leptin levels changes, which confirms the importance of this associative region in eating behaviours. The right and left hippocampus have been found significantly negatively correlated to body weight/leptin changes, which confirms the importance of limbic structures in the eating behaviours and suggests that smaller hippocampal volumes might be associated with increased risk of body weight gain, while leptin elevations might play a role in the weight gain or merely reflect this phenomenon. 

### 5.1. Limitations

This study was limited to the examination of gray matter, and we did not analyse the white matter since we did not have a specific hypothesis about it. Benzodiazepines and antidepressant medications were allowed and could also have been a confounding variable. We worked with total ghrelin measures, which combine both bioactive and inactive forms of ghrelin and could have resulted in a less precise evaluation of this hormone's levels variations associated with olanzapine treatment.

## 6. Conclusion

Our results suggest that modifications of metabolic parameters for patients treated with olanzapine are correlated with different grey matter regions that have already been described as involved in appetite and foodintake control mechanisms. However, these findings are only correlations and further studies are needed in order to understand the meaning and the direction of these associations. These results also should be compared or discussed in line with the data obtained by studies using functional brain imaging paradigms.

## Figures and Tables

**Table 1:** Biometrical, metabolic, and clinical variations during the study.

	Average	Standard deviation
Body Weight (kg)	3,35	5,43
BMI (kg/m^2^)	1,11	1,99
Abdominal circumference (cm)	0,20	1,14
Fasting glucose (mmol/L)	−0,02	0,84
Fasting total cholesterol (mmol/L)	0,20	1,14
Fasting triglycerides (mmol/L)	0,25	0,69
Fasting ghrelin (pg/mL)	−102,18	179,01
Fasting leptin (ng/mL)	1,75	4,14
PANSS global score	−3,94	17,43

**Table 2:** Correlations between grey matter volumes after olanzapine treatment and body weight changes observed in schizophrenia patients.

	R/L	Regions based on AAL atlas	MNI Coordinates			Voxels	*Z*-Score	*P* value
			*X*	*Y*	*Z*			
Positive correlations Body Weight Change and Brain Structures	R	Superior parietal	37	−50	59	1302	2.85	.002
	R	Inferior frontal operculum	50	8	18	1852	2.40	.008

Negative correlations Body Weight Change and Brain Structures	L	Post-central	−52	−11	24	7107	3.32	.0001
	L	Hippocampus	−33	−20	−13	1387	1.99	.023
	R	Post-central	45	−15	34	3501	2.38	.009
	L	Precuneus	−14	−39	69	559	2.11	.018
	L	Thalamus	−7	−23	15	179	1.96	.025
	R	Precuneus	10	−59	43	19	1.68	.048

**Table 3:** Correlations between grey matter volumes and fasting ghrelin levels changes observed in schizophrenia patients after olanzapine treatment.

	R/L	Regions based on AAL atlas	MNI Coordinates			Voxels	*Z*-score	*P* value
			*X*	*Y*	*Z*			
Positive correlations Ghrelin Change and Brain Structures	L	Fusiform	−39	−17	−19	318	2.63	.004
	L	Superior Temporal	−57	−30	16	165	2.41	.008
	R	Inferior Frontal Operculum	50	15	15	27	2.41	.008

Negative correlations Ghrelin Change and Brain Structures	R	Caudate	16	18	12	469	2.58	.005

**Table 4:** Correlations between grey matter volumes and fasting leptin levels changes observed in schizophrenia patients after olanzapine treatment.

	R/L	Regions based on AAL atlas	MNI Coordinates			Voxels	*Z*-Score	*P* value
			*X*	*Y*	*Z*			
Positive correlations Leptin Change and Brain Structures	L	Angular	−27	−56	35	100	2.06	.020
	L	Precentral	−47	3	43	507	2.01	.022
	R	Superior Frontal	18	0	66	152	1.80	.036
	R	Precentral	49	3	26	29	1.75	.040

Negative correlations Leptin Change and Brain Structures	R	Middle Frontal	36	21	23	1459	2.42	.008
	R	Precuneus	9	−52	72	1682	2.27	.011
	R	Hippocampus	41	−21	−15	5796	2.11	.018
	L	Precuneus	−10	−42	69	244	1.76	.039

AAL: Automated Anatomical Labelling; MNI: Montreal Neurological Institute.
